# Into Tibet: An Early Pliocene Dispersal of Fossil Zokor (Rodentia: Spalacidae) from Mongolian Plateau to the Hinterland of Tibetan Plateau

**DOI:** 10.1371/journal.pone.0144993

**Published:** 2015-12-14

**Authors:** Qiang Li, Xiaoming Wang

**Affiliations:** 1 Key Laboratory of Vertebrate Evolution and Human Origins of Chinese Academy of Sciences, Institute of Vertebrate Paleontology and Paleoanthropology, Chinese Academy of Sciences, Beijing, China; 2 CAS Center for Excellence in Tibetan Plateau Earth Sciences, Beijing, China; 3 Department of Vertebrate Paleontology, Natural History Museum of Los Angeles County, Los Angeles, California, United States of America; Team 'Evo-Devo of Vertebrate Dentition', FRANCE

## Abstract

This paper reports the fossil zokors (Myospalacinae) collected from the lower Pliocene (~4.4 Ma) of Zanda Basin, southwestern Tibet, which is the first record in the hinterland of Tibetan Plateau within the Himalayan Range. Materials include 29 isolated molars belonging to *Prosiphneus eriksoni* (Schlosser, 1924) by having characters including large size, highly fused roots, upper molars of orthomegodont type, m1 anterior cap small and centrally located, and first pair of m1 reentrants on opposing sides, high crowns, and high value of dentine tract parameters. Based on the cladistics analysis, all seven species of *Prosiphneus* and *P*. *eriksoni* of Zanda form a monophyletic clade. *P*. *eriksoni* from Zanda, on the other hand, is nearly the terminal taxon of this clade. The appearance of *P*. *eriksoni* in Zanda represents a significant dispersal in the early Pliocene from its center of origin in north China and Mongolian Plateau, possibly via the Hol Xil-Qiangtang hinterland in northern Tibet. The fast evolving zokors are highly adapted to open terrains at a time when regional climates had become increasingly drier in the desert zones north of Tibetan Plateau during the late Miocene to Pliocene. The occurrence of this zokor in Tibet thus suggests a rather open steppe environment. Based on fossils of large mammals, we have formulated an “out of Tibet” hypothesis that suggests earlier and more primitive large mammals from the Pliocene of Tibet giving rise to the Ice Age megafauna. However, fossil records for large mammals are still too poor to evaluate whether they have evolved from lineages endemic to the Tibetan Plateau or were immigrants from outside. The superior record of small mammals is in a better position to address this question. With relatively dense age intervals and numerous localities in much of northern Asia, fossil zokors provide the first example of an “into Tibet” scenario–earlier and more primitive taxa originated from outside of the Tibetan Plateau and the later the lineage became extinct in southwestern Tibet.

## Introduction

Zokors in the family Spalacidae are typical Asian endemic burrowing rodents. Their fossil species were widespread in Neogene strata of northern China, Mongolian Plateau and surrounding areas. Similar to the arvicolines, fossil zokors also have a high turnover rate and have a high potential as key taxa for Neogene biostratigraphic correlations [1]. In China, fossil zokors are normally found in northern parts of the country, but are rare in Qinghai Province in the northern Tibetan Plateau and have never been found in Tibetan Autonomous Region at the southern Tibetan Plateau. To our knowledge, there are only three fossil zokor localities in the Tibetan Plateau. They are all from the northern part of the plateau in Qinghai Province: the Guide Basin yielding *Allosiphneus arvicolinus* (= *Siphneus arvicolinus*)[2,3], the Gonghe Basin yielding *Allosiphneus arvicolinus* (= *Myospalax arvicolinus*) and *Myospalax fontanieri* [3], and the Kunlun Pass Basin producing *Prosiphneus* cf. *P*. *eriksoni* [4]. The zokor site of Kunlun Pass Basin is early Pliocene in age, whereas those from Guide and Gonghe basins are relatively late in age, Early to Middle Pleistocene. Extant zokors on the Tibetan Plateau are also restricted to Qinghai and are notably absent in the hinterland of Tibet. Our discovery of a fossil zokor in Zanda Basin, in the Himalaya Range, is thus the first record of its kind in Tibet and has implications in biochronology, zoogeography, and paleoenvironment.

## Geologic Setting

Zanda Basin is in Ngari District in southwestern Xizang (Tibet) Autonomous Region, China. It is one of the largest late Cenozoic basins on southwestern Tibetan Plateau, and it has a NW-SE orientation reaching ~150 km in its long axis and 20–50 km across. Considering tectonics, Zanda Basin has been postulated to be either a half graben controlled by the Karakorum Fault to the northeast and the South Tibetan Detachment System to the southeast [5,6] or a hybrid supradetachment/strike-slip basin caused by the uplift of the Leo Pargil to the northwest and Gurla Mandhata dome to the southeast [7,8]. Neogene sediments in the basin consist mainly of a series of fluviolacustrine mudstone, siltstone, sandstone and conglomerate with about 800 m maximum thickness. Mammalian remains from the sediments were sporadically reported more than 30 years ago, including a primitive giraffe *Palaeotragus microdon* and a three-toed horse *Hipparion* (*Plesiohipparion*) *zandaense* discovered by geologists during the multidisciplinary expeditions organized by the Chinese Academy of Sciences in 1973–1977 [9,10]. Paleontologic explorations began in 2006 by a joint team of Institute of Vertebrate Paleontology and Paleoanthropology and Natural History Museum of Los Angeles County. Through excavations and screen-washings undertaken during our field seasons in 2006–07, 2009–10 and 2012, the Zanda Basin produced abundant fossil mammals, which have become the richest and most diverse mammal assemblage in the Cenozoic basins on Tibetan Plateau [11,12]. Discoveries of ancestral woolly rhino, snow leopard, and arctic fox from the Zanda Basin inspired the “out of Tibet” hypothesis about the Ice-Age megafauna origins [13,14,15,16,17,18].

**Fig 1:**
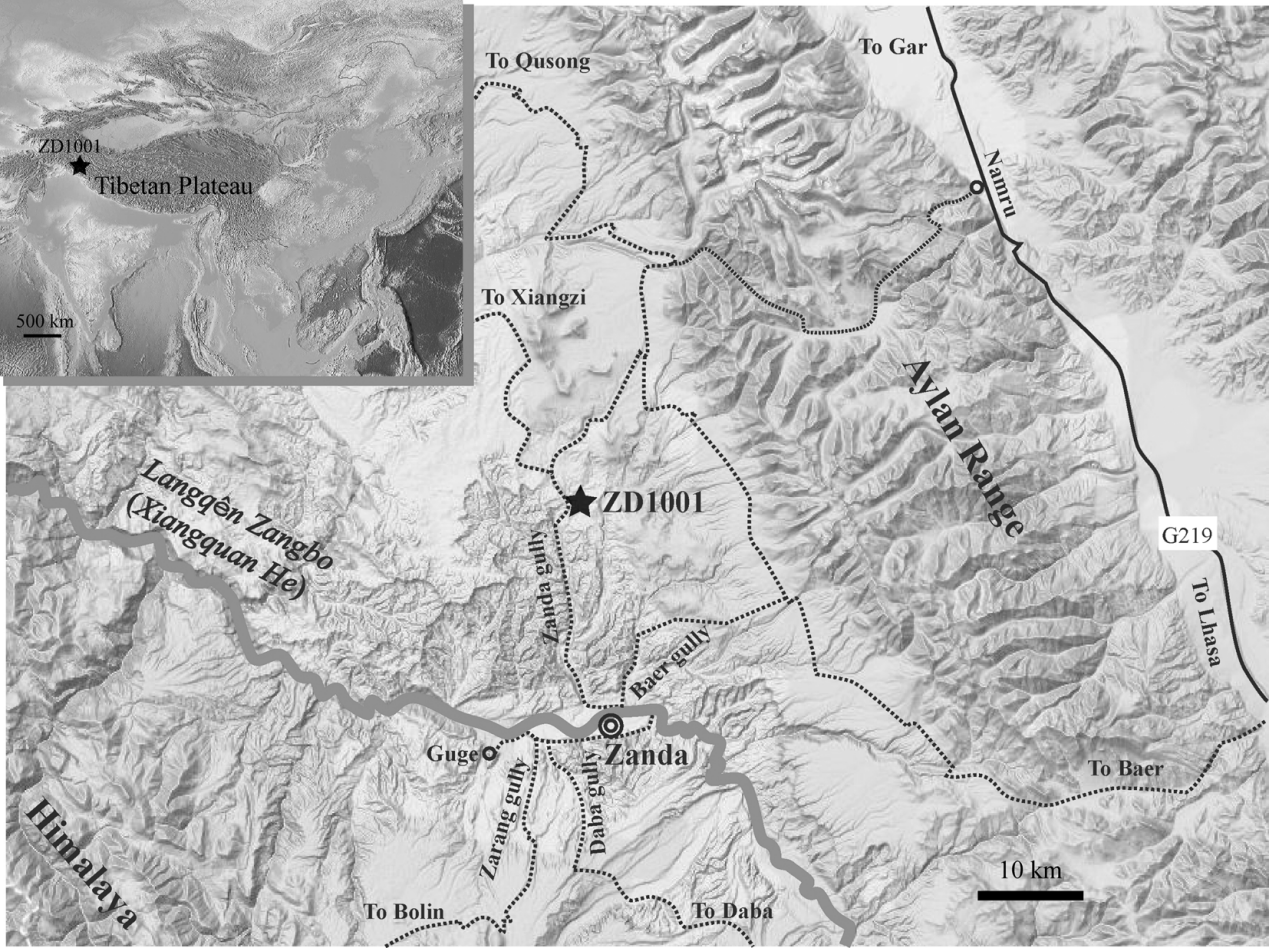
Geographic map of the zokor fossil site, loc. ZD1001 (star), in Zanda Basin, southwestern Tibetan Plateau. Black solid line, national highway; Dashed line, country road; Grey line, river; Circles: County seat and villages.

IVPP ZD1001 locality (31°39'58.2" N, 79°44'57.3" E, ~4114 m above sea level), where the zokor material is associated with ancient snow leopard (*Panthera blytheae*) and arctic fox (*Vulpes zhudingi*), was discovered in August 2010 and located at the east side of Zanda Canyon, north of Langqên Zangbo River (Xiangquan He in Chinese, or an upper tributary of the Sutlej of India and Pakistan), ~20 km northwest of Zanda county seat (Fig 1). The remains are produced from a ~20 cm grey-white or grey-greenish fine sandstone mixed with mud pellets, which overlies a rustic yellowish conglomeratic bed. In lithostratigraphy, the fossil bed can be correlated to the middle lacustrine section of the Zanda Basin [6] and is roughly equivalent to the 335 m level of the South Zanda section in Saylor et al. [19]. The locality ZD1001 bearing fossil zokors was correlated to the GPTS chron C3n.1r, with an age of ~4.4 Ma [12].

## Materials, Methods and Abbreviations

Twenty-eight bags, about a half ton of matrix from the ZD1001 locality were screen-washed in 2010, and hundreds of remains of mammals were collected. Of these 29 isolated molars belong to a fossil zokor.

All specimens (listed below) are housed in the Institute of Vertebrate Paleontology and Paleoanthropology, Chinese Academy of Sciences in Beijing, and are available for examination by qualified researchers. All necessary permits were obtained for the described study, which complied with all relevant regulations.

Terms for dentition and measurement are adopted from Zheng et al. [20]. Teeth were measured with a Wild Heerbrugg microscope to the nearest 0.01 mm. All specimens described in this paper are stored in the Institute of Vertebrate Paleontology and Paleoanthropology.

**Institutional Abbreviations**: **HHPHM**, Huangho-Peiho Museum (or Musée Haong ho Pai ho de Tientsin, predecessor of the present Tianjin Nature Museum); **IVPP**, Institute of Vertebrate Paleontology and Paleoanthropology, Chinese Academy of Sciences; **LMA/S**, Land Mammalian Age or Stage.

**Anatomical Abbreviations**: **ac**, anterior cap; **AL**, anterior lobe; **bra** (**BRA**), labial reentrant angle (lowercase and capital letters for lower and upper molars, respectively); **bsa** (**BSA**), labial salient angle; **L**, maximum length of the wear surface; **lra** (**LRA**), lingual reentrant angle; **lsa** (**LSA**), lingual salient angle; **pl**, posterior lobe; **t** (**T**), triangle; **W**, maximum width of the wear surface; **a**, **b**, **c**, **d**, **e** and **A**, **B**, **C**, **D**, dentine tract parameters [20]. **V**, prefix to vertebrate fossils of IVPP; **ZD**, Zanda Basin, prefix to IVPP field numbers.

### Systematic paleontology

**Order** Rodentia Bowdich, 1821

**Family** Spalacidae Gray, 1821

**Subfamily** Myospalacinae Lilljeborg, 1866

**Genus** *Prosiphneus* Teilhard de Chardin, 1926

#### Type Species

*Prosiphneus licenti* Teilhard de Chardin, 1926[21].

#### Referred Species

*P*. *eriksoni* (Schlosser, 1924) [22]; *P*. *murinus* Teilhard de Chardin, 1942 [23]; *P*. *tianzuensis* (Zheng et Li, 1982) [24]; *P*. *qiui* Zheng, Zhang et Cui, 2004 [20]; *P*. *haoi* Zheng, Zhang et Cui, 2004 [20].

#### Distribution

Early Late Miocene to Early Pliocene, northern and southwestern China.

#### Remark

In the high level taxonomic attribution, both fossil and extant species of zokors belong to a monophyletic group and are often treated as a subfamily Myospalacinae. However, their family attribution is still controversial. In brief, the arguments mainly consist of four opinions. One is the family Siphneidae, which was first mentioned by Teilhard de Chardin and Young [25] and formally established by Leroy (1941) [26] and was also preferred by Teilhard de Chardin [23], Zheng [1], Qiu and Storch [27] and Zheng et al. [20]. The second is the family Myospalacidae erected by Kretzoi [28] (see also Pavlinov and Rossolimo [29]; Rossolimo and Pavlinov [30]). The third is the family Cricetidae, which is mainly supported by Chinese mammalogists and paleontologists on morphologic grounds [31,32,33]. The fourth is the family Spalacidae, which is mainly supported by modern molecular phylogenetic studies [34,35,36]. Zokors are an East Asia endemic group that appeared in the late Middle or early Late Miocene. Fossil evidences generally support a close relationship between zokors and cricetids. The Myospalacinae share some dental characters with Asian Gobicricetodontinae *Plesiodipus*, which was regarded as having a close relationship between them [1,20,33,37,38]. Our recent research on the Neogene rodents of central Nei Mongol also suggests a close relationship between the Myospalacinae and Gobicricetodontinae, and the former may be derived from some form of the latter, but not from *Plesiodipus*. We suggest that zokors evolved in a different way compared to the pathway of *Plesiodipus* [39]. We regard the species *Prosiphneus qinanensis* erected by Zheng, Zhang and Cui in 2004 [20] to be possibly a misidentified taxon, whose holotype (m1, V 14043) and three m2s (V 14044.1–3) should be referred to *Plesiodipus* because of its shorter neck between the anterior cap (ac) and the triangle 2 (t2 or protoconid) on m1, more quadrate buccal salient angle 2s (bsa2s or external angles of protoconids), sharper buccal salient angle 1s(bsa1s or external angles of hypoconids), and shallower buccal reentrant angle 1s (bra1s or ectosinusids) on the m1 and the m2. *Prosiphneus lyratus* Teilhard de Chardin, 1942 was regarded as the type species of genus *Pliosiphneus* first erected by Zheng [1] and recently re-defined by Liu et al. [33]. Significantly, molar characters of the holotype of “*Prosiphneus*” *lyratus* belonging to an old individual (HHPHM 31.076) are still indeterminable. It is difficult to directly compare “*P*.” *lyratus* to the other *Prosiphneus* species established on only isolated teeth. Caution should be exercised in referring any isolated teeth to this species.

**Fig 2:**
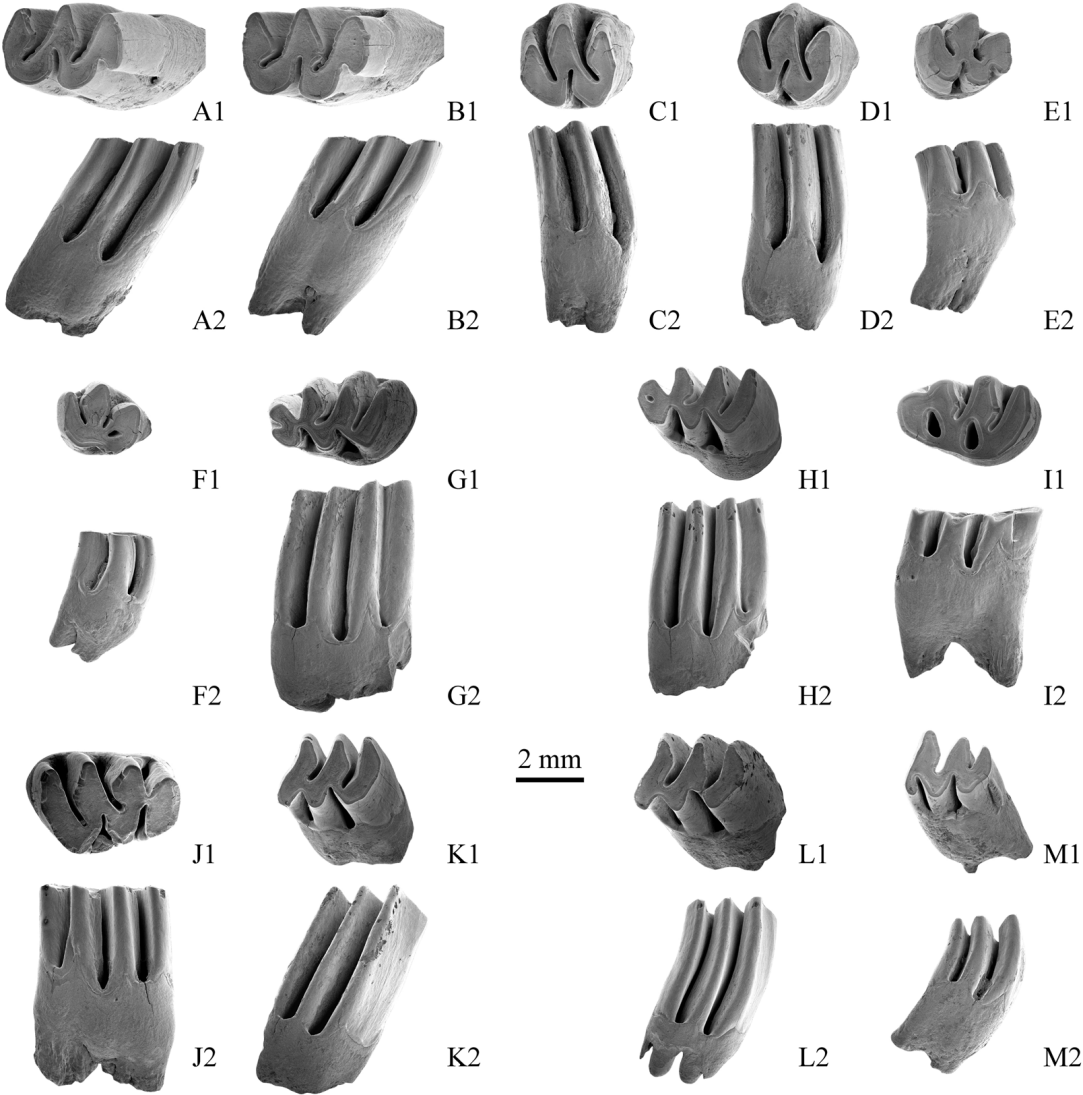
Molars of *Prosiphneus eriksoni* from loc. ZD1001, Zanda, Tibet. A. right M1, V 18032.2; B. right M2, V 18032.3; C. right M2, V 18032.6; D. right M2, V 18032.7; E. left M3, V 18032.10; F. left M3, V 18032.11; G. left m1, V 18032.13; H, left m1, V 18032.14; I, left m1, V 18032.15; J. right m1, V 18032.17; K. left m2, V 18032.19; L. left m2, V 18032.20; M. left m3, V 18032.25. A1-M1, occlusal view; A2-F2, labial view, G2-M2, lingual view, showing the dentine tracts.

*Prosiphneus eriksoni* (Schlosser, 1924)

(Fig 2; Tables 1 and 2)

**Table 1:** Tooth measurements (maximum length and width of the wear surface) of *Prosiphneus eriksoni* from loc. ZD1001, Zanda Basin, Tibetan Plateau (mm).

	Length		Width		
Tooth	Average	Range	Average	Range	Number
M1	3.52	3.37–3.69	2.60	2.39–3.00	4
M2	2.56	2.32–2.82	2.19	1.81–2.49	4
M3	2.36	2.28–2.43	2.02	2.01–2.02	2
m1	3.69	3.41–3.92	2.20	1.90–2.53	5
m2	3.10	2.92–3.25	2.25	1.90–2.65	5
m3	2.37	2.27–2.62	1.93	1.52–2.23	4

**Table 2:** Dentine tract measurements of molars of *Prosiphneus eriksoni* from loc. ZD1001, Zanda Basin, Tibetan Plateau (mm).

Tooth	Dentine tract parameters				
Upper	A	B	C	D	
M1	0–0.22	0.99–1.47	0.99–1.49	0.07–0.36	(Range)
n = 4	0.12	1.23	1.25	0.23	(Mean)
M2	0.31–0.80	0.37–0.99	0.58–1.01	0–0.29	(Range
n = 4	0.57	0.62	0.75	0.10	(Mean)
M3	0–0.17	0–0.16	0.38–0.60	0.10–0.11	(Range
n = 2	0.09	0.08	0.49	0.10	(Mean)
Lower	a	b	c	d	e
m1	0	0.47–0.94	0.63–0.94	0.38–0.58	0–0.46
n = 5	0	0.71	0.79	0.52	0.24
m2	-	0.15–0.59	0.43–0.93	0.30–0.71	0.14–0.33
n = 5	-	0.40	0.66	0.49	0.25
m3	-	0.36–0.60	0.77–0.94	0	0
n = 3	-	0.52	0.85		

*Prosiphneus* cf. *P*. *eriksoni*: Deng et al., 2011: Table S1 [13]

*Prosiphneus* cf. *P*. *eriksoni*: Wang et al., 2013a: 282 [11]

*Prosiphneus* cf. *P*. *eriksoni*: Wang et al., 2013b: 87 [12]

*Prosiphneus* cf. *P*. *eriksoni*: Li et al., 2014 [4]

#### Referred specimens

From locality ZD1001, Zanda Basin, southwestern Xizang (Tibet) Autonomous Region: IVPP V 18032.1–29, 29 isolated teeth including 4 M1s, 5 M2s (2 broken), 3 M3s (1 broken), 6 m1s (1 broken), 6 m2s (1 broken) and 5 m3s (1 broken).

#### Measurements

See Tables 1 and 2.

#### Description

**M1**. The anterior lobe (AL) is the narrowest portion of the tooth with straight anterior border. In occlusal view, the lingual salient angles (LSAs) are semicircular, but the labial ones are rather acute. The lingual reentrant angles (LRAs) are shorter than the labial ones, and they are alternately arranged. The LRA1 and LRA2 are transversely oriented, but the buccal reentrant angles BRA1 and BRA2 stretch posterolingually and reach two third of the wear surface. In lateral view, the dentine tract (DT) is undulating, and all of its peaks overtop the bottom of reentrant angles. The anterior two rootlets are fused, but the posterior one is still separated from them.

**M2**. The outline is typically orthomegodont and similar to that of M1, but without AL. The lingual reentrant is transversely oriented and seated opposite to the labial salient angle. The labial reentrant angles stretch posterolingually. In lateral view, the DT is undulating, and its peaks overtop (rise above) the bottom of reentrants. The rootlets are fused into two wide and flat roots.

**M3**. It is similar to M2 in shape, but the triangle 4 (T4) is smaller than the T1. In the posterolingual side, an extra reentrant is well-developed. It is open (Fig 2: E1) or worn into an islet (Fig 2: F1). In lateral view, the lingual DT is nearly straight and not overtopping the bottom of the reentrants, but the labial DT is undulating and overtops the reentrants. It has two rootlets with groove.

**m1**. The labial salient angles are round, but the lingual ones are acute. The anterior cap (ac) is small and oval, and it is longitudinally symmetrical in young individuals (Fig 2: G1, H1) but slightly posterolabially stretched in old individuals (Fig 2: I1, J1). A distinct anterior syncline is present in fresh teeth (Fig 2: G1), and it is enclosed into an islet in the adult (Fig 2. H1). The islet is completely absent in the old individual (Fig 2: I1, J1). The bra2 and lra3 are oppositely arranged. The lra3 is the shortest reentrant on the tooth surface and nearly absent in old individuals (Fig 2: I1). In lateral view, the DT undulates and overtops the bottom of the reentrants.

**m2**. The tooth has 3 salient and 2 reentrant angles in both labial and lingual sides. In occlusal view, the triangles are distinctly posterolingually oblique, and the lingual reentrants are deeper than the labial ones. In lateral view, the DT is undulating and overtops the bottom of the reentrants.

**m3**. The tooth is similar to m2 in shape but smaller in size. The posterior lobe (pl) is very reduced. The lingual DT is slightly higher than the bottom of reentrants, while the labial one is very low and not touching it. The root is strongly posteriorly curved.

#### Comparison

Teilhard de Chardin and Young [25] divided zokors into two genera, the fossil genus *Prosiphneus* with rooted molars plus the extant genus *Siphneus* (= *Myospalax*) with rootless molars. Based on cranial, especially occipital characters, they further subdivided *Siphneus* into the convex *S*. *fontanieri*, concave *S*. *psilurus* and flat *S*. *tingi* groups. Teilhard de Chardin [23] followed this classification and singled out the molar roots (rooted or rootless), M1 occlusal pattern (ortho- or clinomegodont), sagittal area and occiput morphology as key characters. Zheng [40] first introduced the dentine tract parameters for the fossil rooted zokor, in parallel to the earlier usage for fossil arvicolines. Based on cranial and dental characters, Zheng [1,38] summarized the fossil zokors of East Asia and classified them into 3 subfamilies and 10 genera, while re-defining the dentine tract parameters. However, the classification by Zheng has not been widely accepted with the validity of these taxa still in question [36,41]. More recently, Zheng et al. [20] and Liu et al. [33] emended the measurement method of dentine tracts and proved its validity at species level in the classification of genus *Prosiphneus*, which provides guidance for this study.

**Fig 3:**
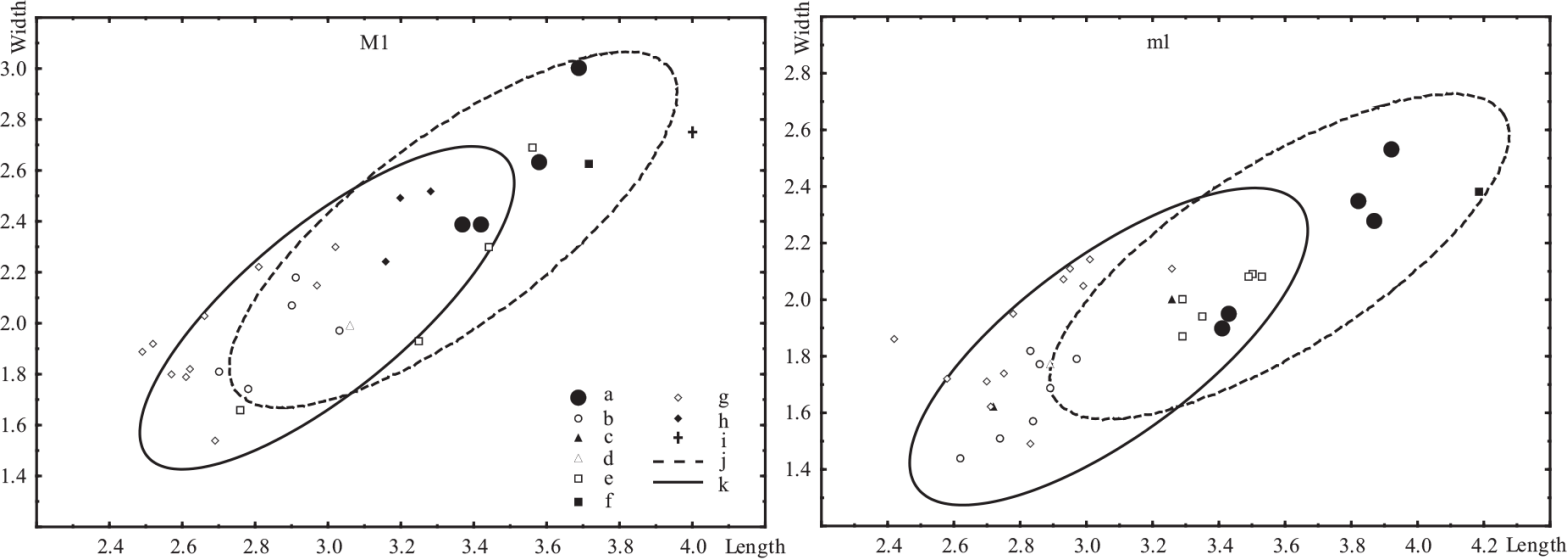
Scatter diagram of the measurements of M1 (left) and m1 (right) of *Prosiphneus* and “*Pliosiphneus*” *lyratus*. a, *Prosiphneus* cf. *P*. *eriksoni*, Zanda, Tibet; b, *P*. *qiui*, Amuwusu, Nei Mongol; c, *P*. *haoi*, Qin’an, Gansu; d, *P*. *licenti*, Qin’an, Gansu; e, *P*. *tianzuensis*, Tianzhu, Gansu; f, *Prosiphneus* cf. *P*. *eriksoni*, Kunlun Pass, Qinghai; g, *P*. *licenti*, Qingyang, Gansu; h, *P*. *murinus*, Yushe, Shanxi; i, “*Pliosiphneus*” *lyratus*, Yushe; j, *Prosiphneus* cf. *P*. *eriksoni*, Bilike, Nei Mongol; k, *P*. *eriksoni*, Ertemte, Nei Mongol.

In the Zanda zokor, the M1 occlusal pattern is orthomegodont type; the anterior cap (ac) on m1 is relatively small, symmetrical and medially seated, and bra2 and lra3 on m1 are oppositely arrayed. According to the diagnosis by Zheng [1,38], Zanda zokor should belong to the convex-skulled group. Zheng [1] considered this group to include *Prosiphneus*, *Myotalpavus*, and *Pliosiphneus* with rooted molars and also *Eospalax* and *Allosiphneus* that have rootless molars. Molars of the Zanda zokor are rooted, which excludes the latter two genera. Among the three genera with rooted molars, the validity of *Myotalpavus* and *Pliosiphneus* are still questionable. McKenna and Bell [41] and Wilson and Reeder [36] both regarded them as synonyms of *Prosiphneus*. Zheng et al. [20] also formally abandoned *Myotalpavus*. *Pliosiphneus*, on the other hand, was erected by Zheng [1], who referred only one species, which was originally described as *Prosiphneus lyratus* by Teilhard de Chardin [23], based on a skull belonging an old individual from Yushe Basin, Shanxi Province (HHPHM 31.076). More recently, Liu et al. [33] redefined the genus *Pliosiphneus* and still assigned “*Prosiphneus*” *lyratus* as its type species. The specimen HHPHM 31.076 is possibly lost. Teilhard de Chardin [23] only measured the length of entire upper dentition but not of individual teeth of HHPHM 31.076. Judging from his figures 36 and 36a, the length and width of M1 in *Pl*. *lyratus* are about 4.0 mm and 2.8 mm, respectively, which are slightly larger than those of Zanda sample (Fig 3). However, its dentine tracts are unrecognizable. The Zanda sample cannot be further compare to it.

The Zanda zokor sample has rooted molars, symmetrical and medially seated ac on m1, oppositely and relative transversely arrayed buccal reentrant angles bra2 and lra3 on m1, and zero-value dentine tract parameter a. These characters are consistent with the diagnosis for convex-skulled group by Zheng [1,38] and more specifically, for *Prosiphneus* as emended by Zheng et al. [20] and Liu et al. [33]. Zheng et al. [20] redefined the genus *Prosiphneus* and attributed seven species to it, including *P*. *licenti*, *P*. *murinus*, *P*. *eriksoni*, and *P*. *tianzuensis*, as well as three newly erected species, *P*. *qinanensis*, *P*. *qiui*, and *P*. *haoi*. Liu et al. [33] insist on this view. As in aforementioned remarks, *P*. *qinanensis* should be excluded from *Prosiphneus*. Moreover, additional materials of *Prosiphneus* have also been reported as *P*. cf. *P*. *eriksoni* from Bilike, central Nei Mongol and Kunlun Pass Basin, Qinghai Province [4,27].

**Fig 4:**
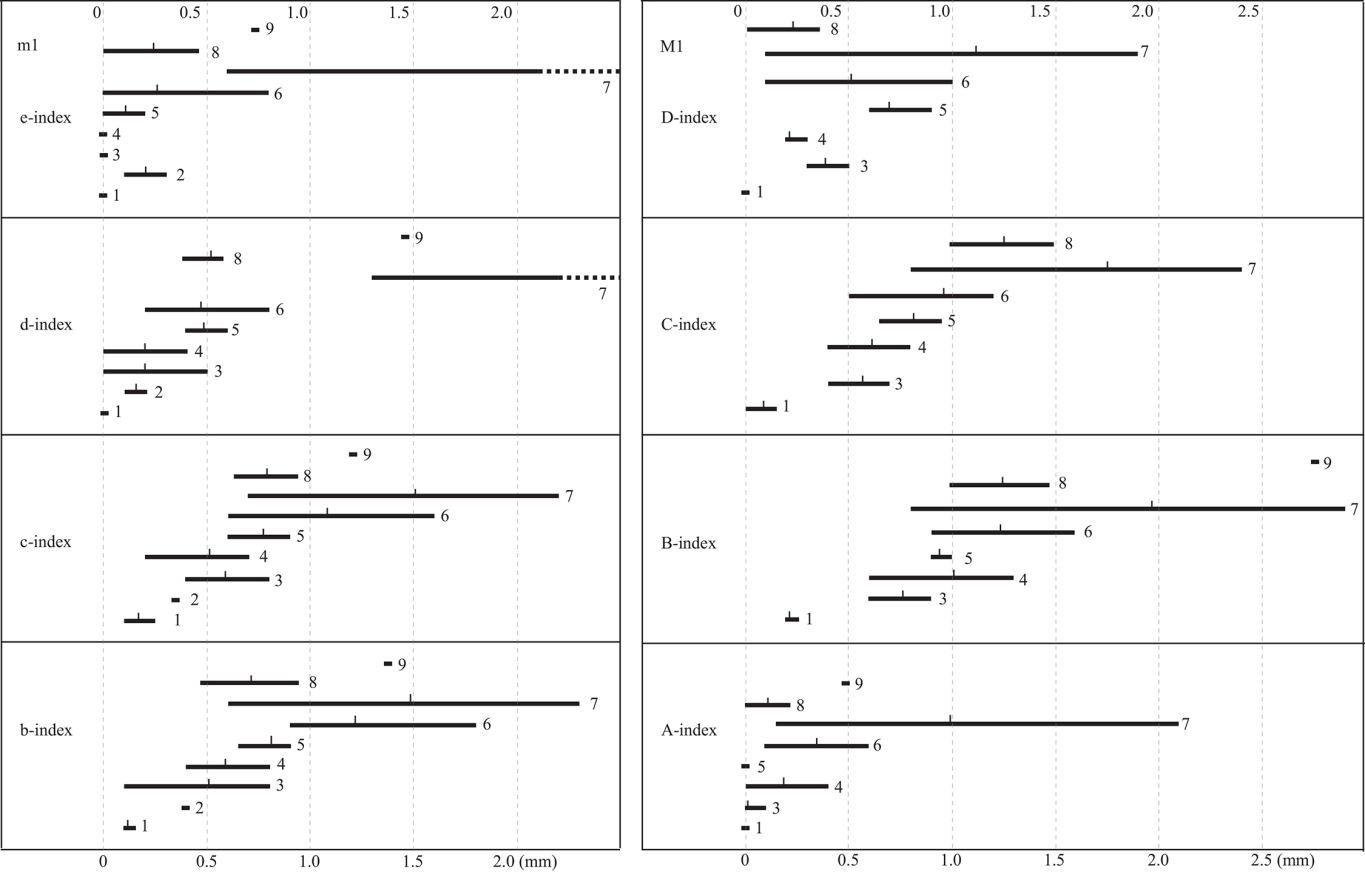
Comparison of the dentine tract measurements of M1 (right) and m1 (left) of *Prosiphneus*. 1, *P*. *qiui*, Amuwusu; 2, *P*. *haoi*, Qin’an; 3, *P*. *licenti*, Qingyang; 4, *P*. *murinus*, Yushe; 5, *P*. *tianzuensis*, Tianzhu; 6and 8 *P*. *eriksoni*, 6, Ertemte; 8, Zanda; 7and 9, *Prosiphneus* cf. *P*. *eriksoni*: 7, Bilike; 9, Kunlun Pass. 1–6, data cited from Zheng et al. (2004: Tables 1 and 2); 7, remeasured and 8, 9 measured by authors.

In size, the Zanda sample is distinctly larger than teeth of *P*. *qiui* from Amuwusu, Nei Mongol and *P*. *haoi* from Qin’an, Gansu, and slightly larger than *P*. *licenti* from Qingyang and Qin’an, *P*. *murinus* from Yushe and *P*. *eriksoni* from Ertemte, Nei Mongol, and close to *P*. *tianzuensis* from Tianzhu, Gansu and *P*. cf. *P*. *eriksoni* from Bilike and Kunlun Pass (Fig 3). In morphology, the Zanda sample differs from the primitive and lower-crowned species *P*. *qiui* and *P*. *haoi* by its remarkably higher crown or higher values of dentine tract parameters, highly fused roots, deeper labial reentrant angles, and rounder salient angles on m1. It also differs from *P*. *licenti* and *P*. *murinus* mainly by having high-value dentine tract parameters on the molars. Zanda sample can be distinguished from *P*. *tianzuensis* by having distinctly higher values of dentine tract parameters on upper teeth, and more symmetrical and medially seated ac on m1. Though of similar size, the Zanda sample differs from *Prosiphneus* cf. *P*. *eriksoni* from Bilike and Kunlun Pass by having lower values of dentine tract parameters on molars (Fig 4). In dental morphology, existing material from Zanda is essentially identical to *Prosiphneus eriksoni* from Ertemte, Nei Mongol. The minor differences between Ertemte and Zanda samples are the former having smaller sized molars with slightly lower b-index of m1 and higher C-index of M1.

### Cladistic analysis and phylogeny of *Prosiphneus*

No previous cladistics relationship exists for the genus *Prosiphneus*. Our own attempt is of limited scope, aimed at elucidating species relationships within the genus, and we exclude some progressive fossil and extant genera not directly relevant in this study. *Democricetodon lindsayi* Qiu, 1996 is selected as an outgroup. Considering the possible close relationships among *Prosiphneus*, *Plesiodipus* and *Gobicricetodon*, a total of 12 taxa including all 7 known species of *Prosiphneus* plus Zanda zokor and *Plesiodipus leei*, *Pl*. *progressus*, *Gobicricetodon flynni* and *G*. *robustus* in Qiu [37] are chosen as ingroups. Owing to the fact that most of the relevant forms are represented by isolated teeth, we thus constructed a character matrix based on teeth only (Tables 3–5).

**Table 3:** Data matrix employed in the cladistic analysis, including 13 taxa and 55 characters. A question mark means that the information is not available. Part 1, characters 1–18.

Taxa	0	1	2	3	4	5	6	7	8	9	10	11	12	13	14	15	16	17	18
*Democricetodon lindsayi*	0	0	0	0	3	1	0	0	0	2	1	0	1	0	0	0	0	1	0
*Plesiodipus leei*	0	1	1	1	1	1	2	1	1	2	0	2	0	0	1	2	0	0	2
*Plesiodipus progressus*	1	2	1	1	1	1	2	1	1	2	0	2	0	1	1	3	0	0	2
*Gobicricetodon flynni*	0	2	0	1	0	0	1	0	0	0	0	1	1	2	1	0	0	1	1
*Gobicricetodon robustus*	0	3	1	1	0	0	1	0	0	1	0	1	1	2	1	0	0	0	2
*Prosiphneus qiui*	1	2	1	1	2	1	2	0	1	2	0	2	1	3	1	1	1	1	3
*Prosiphneus haoi*	1	3	1	1	?	?	?	?	?	?	?	?	?	?	?	?	?	?	3
*Prosiphneus licenti*	1	2	2	1	2	1	2	0	1	2	1	3	1	3	2	3	1	1	3
*Prosiphneus murinus*	1	3	2	1	2	1	2	0	1	2	1	3	1	3	2	3	2	1	3
*Prosiphneus tianzuensis*	1	4	2	1	2	1	2	0	1	2	1	3	1	3	2	3	3	1	3
*P*. *eriksoni* (Ertemte)	1	4	2	1	2	1	2	0	1	2	1	3	1	3	2	3	3	2	3
*P*. *eriksoni* (Zanda)	1	4	2	1	2	1	2	0	1	2	1	3	1	3	2	3	3	2	3
*Prosiphneus* cf. *P*. *eriksoni*	1	4	2	1	2	1	2	0	1	2	1	3	1	3	2	3	3	2	3

**Table 4:** Data matrix employed in the cladistic analysis, including 13 taxa and 55 characters. A question mark means that the information is not available. Part 2, characters 19–36.

Taxa	19	20	21	22	23	24	25	26	27	28	29	30	31	32	33	34	35	36
*Democricetodon lindsayi*	0	0	0	0	0	0	2	1	0	1	0	0	0	1	0	2	0	2
*Plesiodipus leei*	2	2	2	1	2	0	0	0	1	0	0	0	0	1	2	1	0	0
*Plesiodipus progressus*	2	3	2	1	3	0	0	0	1	1	?	0	?	1	2	1	0	0
*Gobicricetodon flynni*	1	1	1	0	0	0	1	0	0	1	0	0	0	0	1	0	0	0
*Gobicricetodon robustus*	1	1	2	0	0	0	0	?	0	1	0	0	0	0	1	0	0	1
*Prosiphneus qiui*	2	2	3	2	1	1	1	0	1	1	0	1	0	1	3	1	1	1
*Prosiphneus haoi*	2	3	3	2	3	2	?	?	?	?	?	?	?	1	3	2	1	2
*Prosiphneus licenti*	2	3	3	2	3	2	3	1	1	1	1	2	1	1	3	2	1	2
*Prosiphneus murinus*	2	3	3	2	3	2	3	1	1	1	1	2	1	1	3	2	1	2
*Prosiphneus tianzuensis*	2	3	3	2	3	3	3	1	1	1	1	2	1	1	3	2	2	2
*P*. *eriksoni* (Ertemte)	2	3	3	2	3	3	3	1	1	1	1	2	1	1	3	2	3	2
*P*. *eriksoni* (Zanda)	2	3	3	2	3	3	3	1	1	1	1	2	1	1	3	2	3	2
*Prosiphneus* cf. *P*. *eriksoni*	2	3	3	2	3	3	3	1	1	1	1	2	1	1	3	2	3	2

**Table 5:** Data matrix employed in the cladistic analysis, including 13 taxa and 55 characters. A question mark means that the information is not available. Part 3, characters 37–54.

Taxa	37	38	39	40	41	42	43	44	45	46	47	48	49	50	51	52	53	54
*Democricetodon lindsayi*	1	1	1	0	0	1	0	0	0	0	0	0	0	0	0	0	0	0
*Plesiodipus leei*	1	0	2	1	1	1	0	1	0	2	1	1	1	0	0	0	0	0
*Plesiodipus progressus*	1	0	2	1	1	1	0	1	0	2	1	1	1	0	1	1	1	0
*Gobicricetodon flynni*	0	0	0	0	0	0	0	0	0	1	1	1	0	0	0	0	0	0
*Gobicricetodon robustus*	1	0	0	0	0	0	0	0	0	1	1	1	0	0	0	0	0	0
*Prosiphneus qiui*	1	0	0	0	2	1	1	2	1	2	2	1	2	1	1	0	0	1
*Prosiphneus haoi*	1	1	2	0	2	1	1	2	2	2	2	1	2	2	1	0	0	1
*Prosiphneus licenti*	1	1	2	0	2	1	1	2	2	2	2	1	2	2	1	0	0	1
*Prosiphneus murinus*	1	1	2	0	2	1	1	2	2	2	2	1	2	2	1	0	0	1
*Prosiphneus tianzuensis*	1	1	2	0	2	1	0	2	3	2	2	1	2	3	2	0	0	2
*P*. *eriksoni* (Ertemte)	1	1	2	0	2	1	1	2	3	2	2	1	2	3	2	0	0	2
*P*. *eriksoni* (Zanda)	1	1	2	0	2	1	1	2	3	2	2	1	2	3	2	0	0	2
*Prosiphneus* cf. *P*. *eriksoni*	1	1	2	0	2	1	1	2	3	2	2	1	2	3	2	0	0	2

Cladistic analysis was performed using TNT (ver. 1.1, March, 2015) by Goloboff et al. [42]. Our 13 taxa by 55 characters (see S1 Appendix) data matrix was generated using the Mesquite ver. 3.02 (build 681) [43]. Implicit Enumeration (IE, or branch-and-bound) search method under non-additive assumption was used to obtain an exact solution. The IE recovered 4 equally most parsimonious trees. The strict consensus tree has a length of 122 steps, with Consistency Index = 0.869 and Retention Index = 0.888.

**Fig 5:**
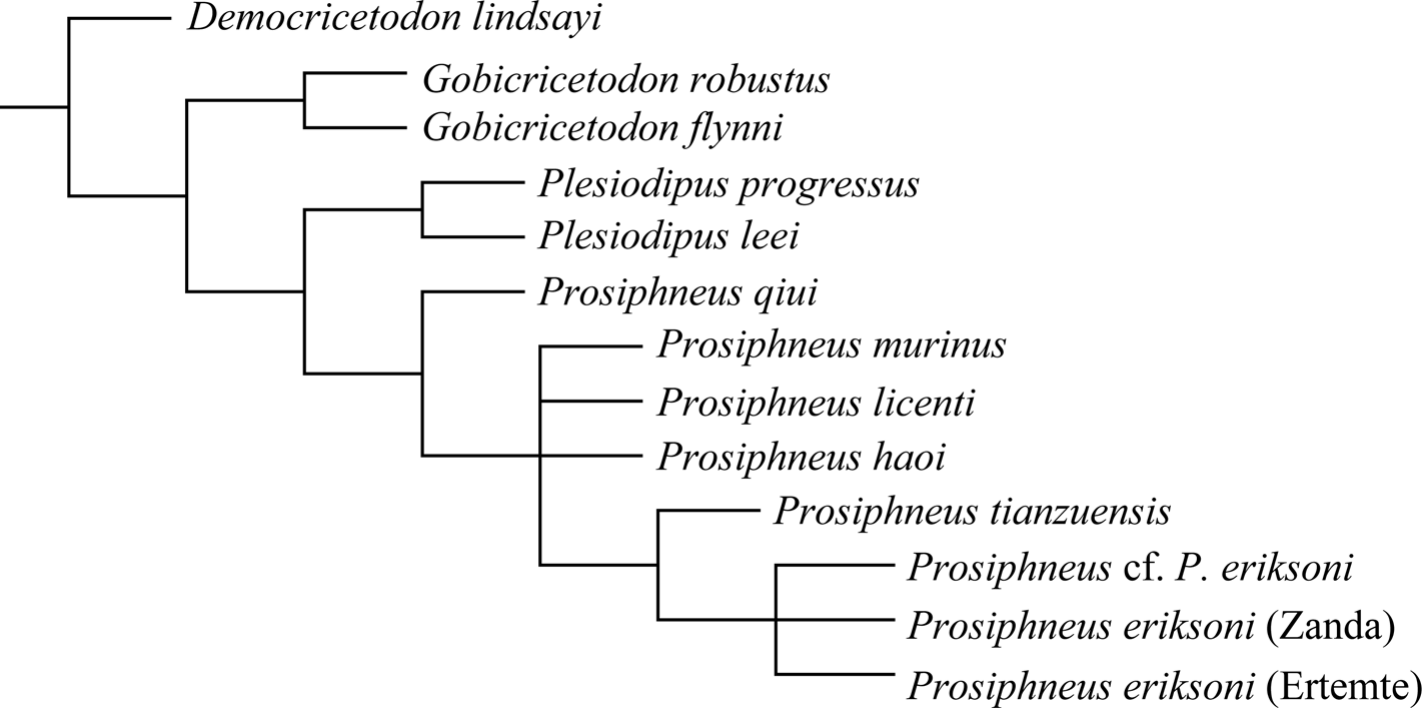
Strict consensus tree (L = 122; CI = 0.869; RI = 0.888) based on the dental character matrix in Table 3, showing the relationships of *Prosiphneus* and relevant taxa.

According to the strict consensus tree (Fig 5), all seven species of *Prosiphneus* and *P*. *eriksoni* of Zanda form a monophyletic clade. *Plesiodipus leei*+*Pl*. *progressus* form a clade of its own and is the sister group of *Prosiphneus*. *Gobicricetodon robustus*+*G*. *flynni* is a more basal clade outside the *Plesiodipus*+*Prosiphneus* group. Within *Prosiphneus*, relationship among *P*. *murinus*, *P*. *licenti*, and *P*. *haoi* is unstable, and similarly within-species relationship of *Prosiphneus* cf. *P*. *eriksoni* of Bilike, *P*. *ericksoni* of Zanda and Ertemte is not fully resolved. These unresolved nodes notwithstanding, a basic relationship emerges that *P*. *qiui* is the most primitive species, and after several transitional species, *P*. *eriksoni* of Zanda is a terminal taxon of *Prosiphneus*.

The above phylogeny is fundamentally consistent with paleontological records. The oldest species of *Prosiphneus* is still *P*. *qiui* from Amuwusu, Nei Mongol, with an age of early Late Miocene or lower/earlyChinese Bahean LMS/A, or equivalent to the European MN9 [44]. *P*. *haoi* was recovered from Qin’an, Gansu, and with a paleomagnetic age of about 8.2–9.5 Ma [20]. *P*. *licenti* has been found from *Hipparion* Red Clay of Qingyang and Qin’an, Gansu, with an age for the former as late Late Miocene Baodean LMS/A and for the latter in the range of 6.5–7.6 Ma by paleomagnetic determination [20,23]. *P*. *murinus* was only discovered from Yushe Basin, Shanxi with an age range of 6.3–4.5 Ma estimated by Zheng [20]. Zheng’s estimation for late occurrences of the species was based on incorrect stratigraphic placement of localities; the latest occurrence of *P*. *murinus* may be no younger than 5.5 Ma (Prof. Lawrence Flynn, personal communication). *P*. *tianzuensis* was only found from Tianzhu, Gansu and restricted to late Late Miocene (roughly equivalent to European MN12) [24]. *P*. *eriksoni* was typically discovered from Ertemte, Nei Mongol, and regarded as latest Miocene and around 5.3 Ma. [45,46]. Moreover, *P*. *eriksoni* from Lingtai, Gansu Province, occurs in the Early Pliocene with a paleomagnetic date of ~4.9 Ma [47]. *P*. *eriksoni* from Zanda, Tibet appears to be in slightly younger age with a paleomagnetic date of ~4.4 Ma [11].

**Fig 6:**
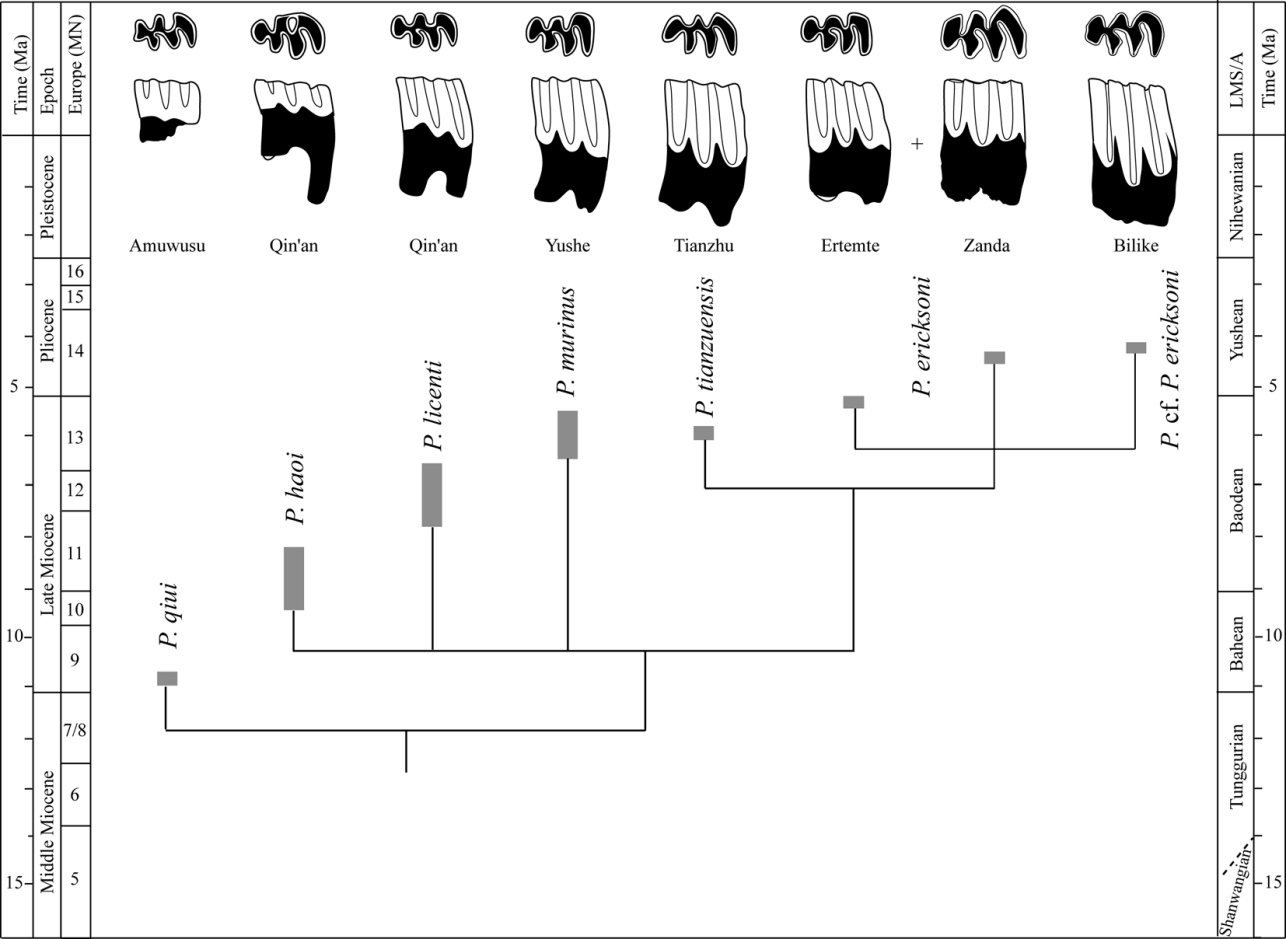
Species ranges and cladistic phylogeny among the *Prosiphneus* in the Neogene China. Most of the species ranges are approximate. The first lower molars are scaled to their approximate relative size and the Fig also shows the m1 evolutionary trends of *Prosiphneus* especially on the heightening of the crown and lateral dentine tracts.

*Prosiphneus* cf. *P*. *eriksoni* was present in the Bilike Fauna, Nei Mongol, and in Kunlun Pass Basin of northern Tibetan Plateau. The Bilike Fauna was regarded as early Pliocene in age and slightly older than the lower Gaotege Fauna yielding the more progressive, concave-skulled zokors with paleomagnetic date of ~4.2Ma [27,46,48,49], whereas the Kunlun Pass Basin fauna was suggested to be about 4.2 Ma in age by lithostratigraphic and magnetic correlation [4]. Liu et al. [33] referred *Prosiphneus* cf. *P*. *eriksoni* from Bilike described by Qiu et Storch [27] to “cf.” *Pliosiphneus lyratus*. Due to a lack of cranial materials, it is difficult to match the isolated teeth from Bilike and Kunlun Pass with the holotype of “*Prosiphneus*” *lyratus* from Yushe. We are inclined to treat the Bilike and Kunlun Pass zokors as a progressive member of *Prosiphneus*. We partly agree with the opinion of Zheng et al. [20] that the lineage of *Prosiphneus* possibly began at *P*. *qiui* and ended in *P*. cf. *P*. *eriksoni*. The evolutionary tendency of this lineage includes increasing size, more fusion of roots, heightening of tooth crown, and increasing values of dentine tract parameters. Among known species of *Prosiphneus*, *P*. *eriksoni* from Zanda has a large size, high degree of root fusion, and high values of dentine tract parameters. It thus is likely to be near the terminal end of the *Prosiphneus* evolutionary lineage, as is also consistent with its younger age (Fig 6).

### Zoogeography

**Fig 7:**
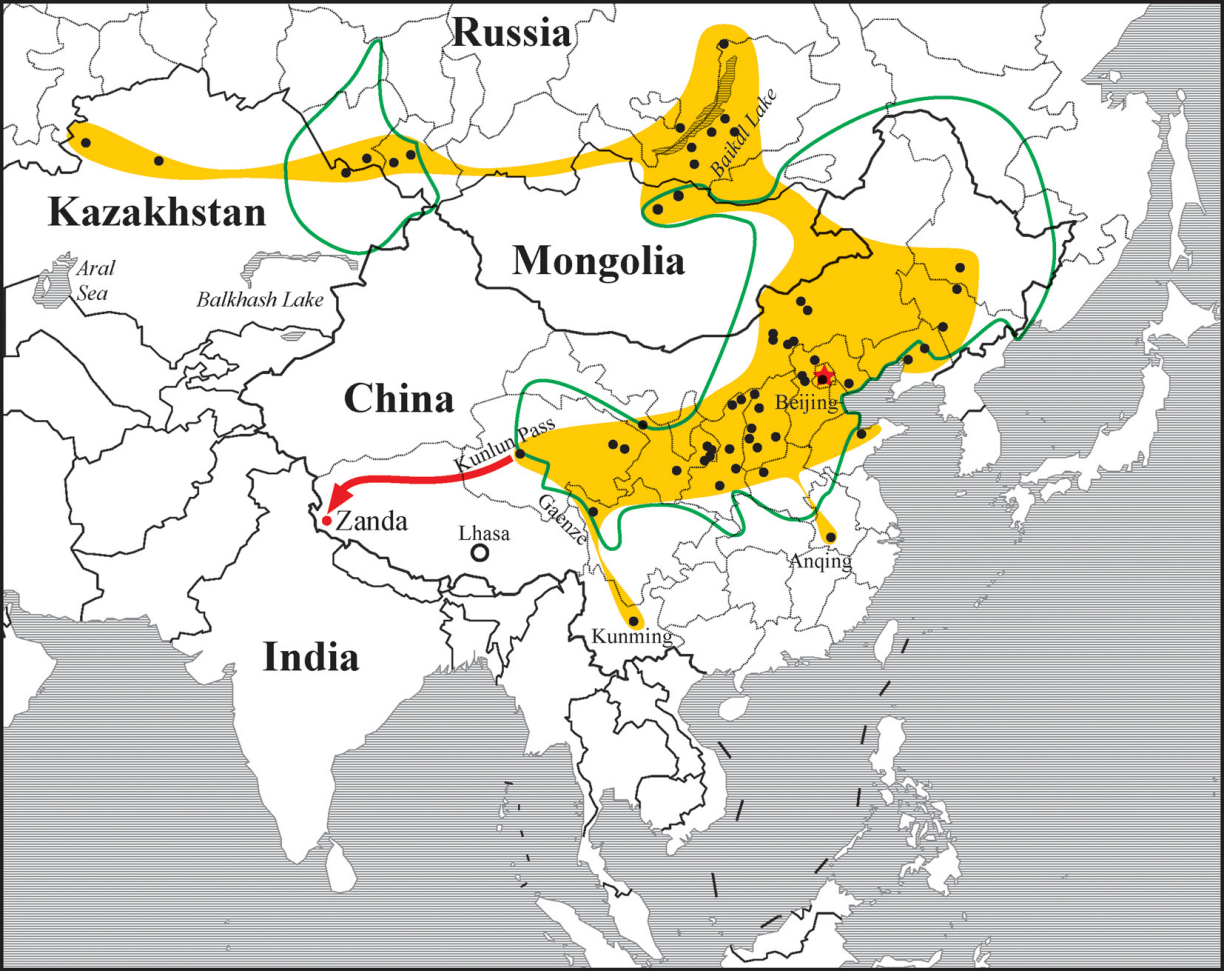
Geographic distribution of the main localities of the fossil and extant zokors. Green line–extant (referred from website of IUCN, http://www.iucnredlist.org/details/14116/0, /14118/0, /14119/0, /14120/0, /14121/0, and /14122/0, accessed on Dec. 11^th^, 2014); yellow shadow and black circles–fossils (based on Zheng, 1994). Red arrow shows a potential dispersal way of the *Prosipheus* from its center of origin in north China and Mongolian Plateau to the Zanda Basin of southwestern Tibetan Plateau, possibly via the Hol Xil-Qiangtang hinterland in northern Tibet.

The extant zokors are Asiatic native burrowing rodents ranging from Kazakhstan, Russian Siberia, northern China, and eastern Mongolia to Korean peninsula [31]. Chinese mammalogists agreed that the living zokors included only one genus *Myospalax* Laxmann, 1769. In China, *Myospalax* mainly lives in the Palearctic region in eastern and northern China, including Inner Mongolia-Xinjiang and northeastern Tibetan Plateau (Qinghai), and it is also present in the transition zones between Oriental and Palearctic regions such as central and southwestern China [31,50,51]. On the Tibetan Plateau, it is only present in its eastern edge where the East Kunlun Mountain south of Golmud is its geographic western-most reach [50], extant zokors do not reach the hinterland of Tibet [52,53]. Fossil zokors had a slightly larger distribution than the extant ones (Fig 7). Their west-most distribution reaches the western border of Kazakhstan, whereas their north–most records can be found north of the Lake Baikal, and the southeastern–most limit nearly reaches the south bank of Yangtze River (Anqing, Anhui Province) [1,54]. Fossil discoveries from Kunming in Yunnan, Gaenze in Sichuan, and Kunlun Pass in Qinghai, were once the closest to Tibet Autonomous Region that had been known [4,55,56]. Our discovery of *Prosiphneus eriksoni* in the Zanda Basin expands the geographic distribution of zokor into the far southwestern corner of Tibetan Plateau, a remarkable expansion with zoogeographic and paleoenvironmental implications.

Current fossil evidence strongly supports that zokors originated from northern China in latest Middle or earliest Late Miocene and had a rapid dispersal and differentiation during Late Miocene and Early Pliocene [1,20,37,38,39]. The presence of *Prosiphneus eriksoni* in the Zanda Basin undoubtedly indicates an Early Pliocene successful dispersal event. It is noteworthy that fossil zokor is also present in the lower Pliocene of the Kunlun Pass Basin reported as *Prosiphneus* cf. *P*. *eriksoni*, and the extant zokors also live in this area [4,50]. Studies of fossil schizothoracine fishes from the Kunlun Pass Basin indicated that the East Kunlun Mountain had not yet become a prominent biological barrier in the early Pliocene [57]. The presence of *Prosiphneus* in Zanda and Kunlun Pass basins indicates a communication between the Tibetan Plateau and North China in early Pliocene. The Kunlun Pass and Zanda basins share *Prosiphneus*, *Nannocricetus*, *Aepyosciurus*, *Ochotona* and *Vulpes zhudingi* [4]. It is obvious that the mammalian interchange between Zanda and Kunlun Pass basins have been rather active in early Pliocene, and the vast and relatively flat terrains in Hoh Xil and Qiangtang basins seem to be suitable grounds for the zokor dispersal on Tibetan Plateau.

In the Zanda Basin modern ecosystem, *Marmota himalayana*, *Lepus oiostolus* and *Ochotona curzoniae* are dominant species of small mammals [52,53]. In the Pliocene of Zanda Basin, lagomorphs are likewise predominant both in species diversity and numbers of individuals. As the largest extant Tibetan rodent, marmots also burrow deep underground and have long hibernations as an adaptation to bitterly cold climates. Interestingly, a recent study by Polly et al. [58] suggests that the marmots also spread into the Tibetan Plateau from a lineage in Siberia during the Pleistocene. Unfortunately no trace of fossil *Marmota* is seen in Pliocene Zanda Basin. However, we discovered the zokor, *Prosiphneus*, and a sciurid, *Aepyosciurus* [12]. The latter is characterized by its highly lophodont and hypsodont cheek teeth, which was regarded as a specialized ground squirrel in Tibetan Plateau [59]. Dental morphology implies that both of them should be adaptive for a steppe environment. We speculate that both *Prosiphneus* and *Aepyosciurus* shared a niche similar to that of modern *Marmota* on Tibetan Plateau.

In recent years, we have formulated an “out of Tibet” hypothesis based on occurrences of ancestral Tibetan large mammals in the Pliocene that appear to have given rise to the Pleistocene megafauna [13,14,15,17]. Fossil records for these large mammals, however, are still far too scarce to permit an evaluation of where these animals ultimately have come from. For most of the taxa, it is not clear whether they evolved from lineages endemic to the Tibetan Plateau, as apparently was the case for the Tibetan antelope (*Qurliqnoria*-*Pantholops* clade) [13,16,60], or they were immigrants from outside of the Tibetan Plateau.

It is thus fortunate that the far more abundant fossil materials of zokors, with their densely sampled age intervals and excellent geographic coverage, provide a clear example of an “into Tibet” scenario, with earlier and ancestral forms originating from outside of the Tibetan Plateau and a nearly terminal species ending in southwestern Tibet. In addition, the richly documented history of Zokors indicates a lineage that is fast evolving and highly adapted to open terrains at a time when regional climates had become increasingly drier in the desert zones north of Tibetan Plateau during the late Miocene to Pliocene as reflected by large mammal communities [61,62]. Physical forcing may have been a predominant mechanism in producing evolutionary novelties in “species factories” that developed under harsh environmental conditions [16,63]. It is perhaps no coincidence that zokors were more responsive to these challenges and became the only documented example of an “into Tibet” lineage. Zanda small mammal records above locality ZD1001 are still very sketchy despite well exposed sections that continue into the Pleistocene. We hope future works will reveal when this great lineage of fossorial rodents had met its ultimate demise–it would be intensely interesting if it became extinct during the onset of the Ice Age.

### Paleoenvironment

Extant zokors are adapted to a multitude of ecological systems such as temperate plain, grassland, farmland, forest edge, and alpine meadow. Their elevation range is also variable. For example, *Myospalax fontanieri* from Sichuan and *M*. *baileyi* from Qinghai are in high mountain areas with elevation range of 2500–4200 m and 2800–4200 m, respectively [64,65]. The elevation of Zanda Basin sediments ranges from 3,700–4,500 m, not significantly different from highest elevation distributions of modern zokors. In this case, the fossil zokors of the Zanda Basin probably do not offer useful constraints to the paleoelevation. On the other hand, hypsodonty in mammalian cheek teeth is usually regarded as an indicator for hard and rough food. In the Zanda Basin, lagomorphs (*Trischizolagus* and *Ochotona*), squirrels (*Aepyosciurus*), high-crowned cricetid, and *Prosiphneus* are all hypsodont taxa. Their co-occurrence therefore indicates a rather arid environment in the Pliocene Zanda Basin. Large ungulates, such as *Hipparion*, *Coelodonta*, some bovids and cervids, were probably adapted for open steppe. The appearances of *Coelodonta thibetana*, *Panthera blytheae* and *Vulpes zhudingi*, in particular, also indicate cold winters with heavy snow covers [13,14,15].

## Conclusion

1. A primitive fossil zokor was discovered in the Zanda Basin, Tibet, which was also the first occurrence of zokors on the hinterland of Tibetan Plateau.
2. Based on the size and morphology, especially dentine tract parameters of molars, the Zanda zokor is referred to *Prosiphneus eriksoni* compared with the form from latest Late Miocene Ertemte Fauna, Nei Mongol.
3. *Prosiphneus eriksoni* of Zanda has large size, highly fused roots, and high crown (or high dentine tract parameters), and should be placed nearly on the terminal end of the *Prosiphneus* lineage based on the cladistic analysis.
4. Occurrence of *Prosiphneus* in Zanda Basin represents a significant dispersal of fossil zokors in early Pliocene. The Hoh Xil and Qiangtang basins are speculated to be the dispersal route.
5. Occurrence of *Prosiphneus* and the other small mammals with high-crowned cheek teeth from ZD1001 indicates an open steppe environment, which is also supported by the appearance of abundant large herbivorous mammals.

## Supporting Information

S1 Appendix List of dental characters used in the phylogenetic analysis. **The character numbers are the same as the data matrix (Tables 3–5)**.
(DOCX)
