# Supplementary material for: Into Tibet: An Early Pliocene Dispersal of Fossil Zokor (Rodentia: Spalacidae) from Mongolian Plateau to the Hinterland of Tibetan Plateau
Source: PLoS One. 2015 Dec 14;10(12):e0144993. doi: 10.1371/journal.pone.0144993 (PMC4678110; doi:10.1371/journal.pone.0144993)
Supplement: S1 Appendix — The character numbers are the same as the data matrix (Tables 3–5). (DOCX) [file pone.0144993.s001.docx]

**S1 Appendix**. List of dental characters used in the phylogenetic analysis. The character numbers are the same as the data matrix (Table 3).

1. Molar occlusal pattern

0: bunodont-lophodont; 1: lophodont

1. Molar size (m1 average length)

0: 1.5 mm ≤ length < 2.0 mm; 1: 2.0mm ≤ length < 2.5 mm; 2: 2.5 mm ≤ length < 3.0 mm; 3: 3.0 mm ≤ length < 3.5 mm; 4: 3.5 mm ≤ length < 4.0 mm

1. Crown height of molars

0: brachydont; 1: semi-hypsodont; 2; hypsodont

1. M1-3 protolophule I

0: present; 1: absent

1. M1 antercone

0: single, buccally seated and with lingually extending ridge; 1: single, small and longitudinally seated; 2: single, wide and flat; 3: slightly bifid

1. M1 protolophule

0: double; 1: single

1. M1 spur of anterolophule

0: present as a distinct spur; 1: present but very weak; 1: absent

1. M1 protocone and hypocone lingual borders after wear

0: round; 1: quadrate

1. M1 protolophule II

0: slightly posteriorly connected to posterior arm of protocone; 1: short and anterolingually fused into posterior arm of protocone

1. M1 paracone spur

0: infrequently present as weak tuber; 1: frequently present as spur; 2: absent

1. M1 mesocone

0: reduced; 1: absent

1. M1 mesoloph

0: often present and long; 1: infrequently present and short; 2: infrequently present and very weak; 3: absent

1. M1 anterosinus

0: open wide and less posteriorly curved; 1: posteriorly strongly curved and relatively narrow

1. M1 protosinus

0: wide and shallow; 1: narrow and shallow; 2: narrow and deep; 3: wide and deep

1. M1 sinus

0: wide and nearly transverse; 1: wide and slightly anteriorly curved; 2: deep and slightly posteriorly curved

1. M1 posterosinus

0: frequently present as islet; 1: infrequently present as islet in young individuals; 2: present as a very shallow groove; 3: absent

1. M1 buccal dentine tract

0: smooth and far below the bottom of valleys; 1: slightly undulant, but far below the bottom of valleys; 2: undulant and nearly reaches or slightly overtop the bottom of valleys; 3: strongly undulant and intensively overtop the bottom of valleys

1. M1 number of roots

0: 4 roots; 1: 3 roots; 2: roots fused

1. M2 lingual branch of anteroloph

0: highly frequently present and long; 1: infrequently present and distinct; 2: infrequently present and very weak; 3: absent

1. M2 protolophule II

0: posteriorly connected to posterior arm of protocone; 1: nearly transversely connected to posterior arm of protocone; 2: transversely fused into posterior arm of protocone

1. M2 mesoloph

0: often present and long; 1: often present but short; 1: infrequently present and very weak; 2: absent

1. M2 protosinus

0: always present as valley; 1: infrequently present as valley; 2: infrequently present as shallow groove; 3: absent

1. M2 anterosinus

0: posteriorly curved and transversely opened; 1: nearly posterolingually directed; 2: nearly transverse

1. M2 posterosinus

0: highly frequently present as islet; 1: infrequently present as islet in young individuals; 2: presents as a very shallow groove; 3: absent

1. M2 buccal dentine tract

0: smooth and far below the bottom of valleys; 1: slightly undulant, but far below the bottom of valleys; 2: undulant and nearly reaches or slightly overtop the bottom of valleys; 3: strongly undulant and intensively overtop the bottom of valleys

1. M2 number of roots

0: 4 roots; 1: mostly 3 roots and few with 4 roots; 2: 3 roots; 4: roots fused

1. M3 ectoloph of paracone

0: present; 1: absent

1. M3 mesoloph

0: long; 1: absent

1. M3 anterosinus

0: strongly posterolingually extended; 1: slightly posterolingually or nearly transversely extended

1. M3 posterosinus

0: present; 1: reduced as a small pit in young individuals

1. M3 buccal dentine tract

0: smooth and far below the bottom of valleys; 1: slightly undulant, but far below the bottom of valleys; 2: undulant and nearly reaches or slightly overtops the bottom of valleys

1. M3 number of roots

0: 3 roots; 1: roots fused

1. m1-2 mesoconid

0: reduced or weak; 1: absent

1. m1-2 mesolophid

0: often present and long; 1: often present and reduced; 2: infrequently present and very weak; 3: absent

1. m1-2 ectomesolophid

0: reduced and frequently present; 1: weak and infrequently present; 2: absent

1. m1 anteroconid

0: small cusp with buccally extending anterolophid; 1: small, lophodont with concaved anterior wall or round cusped; 2: large but anterolingual-posterobuccally entrenched; 4: large and elliptic or round

1. m1 anterolophulid

0: double and anteriorly converging; 1: double and nearly parallel; 2: single

1. m1 spur of anterolophulid

0: occasionally present; 1: absent

1. m1 connection between metaconid and anteroconid

0: connected through lingual anterolophulid; 1: disconnected, metaconid fused with anterolophulid

1. m1 connection between metaconid and protoconid

0: connected through continuous or discontinuous metalophid; 1: disconnected, metalophid transversely connected to anterolophulid; 2: disconnected, metaconid fused with anterolophulid

1. m1 protoconid buccal border after wear

0: round; 1: quadrate

1. m1 posterior arm of protoconid or anterior ectoloph

0: long and normally wide; 1: long and intensively thickened; 2: short and slightly thickened

1. m1 posterior arm of hypoconid

0: very weak; 1: absent

1. m1 anterosinusid and protosinusid

0: slightly obliquely opposite; 1: nearly transversely opposite

1. m1 sinusid

0: wide and nearly rectangular or subtriangular; 1: nearly enclosed and subtriangular; 2: deep and anterolingually directed

1. m1 lingual dentine tract

0: smooth and far below the bottom of valleys; 1: slightly undulant, but far below the bottom of valleys; 2: undulant and nearly reaches or slightly overtop the bottom of valleys; 3: strongly undulant and intensively overtop the bottom of valleys

1. m2-3 anteroconid and anterolophid

0: not fused; 1: fused into a long and slim loph; 2: fused into a short but strong loph

1. m2-3 anterolophid, anteroconid and metaconid

0: not fused; 1: fused into a transverse or slightly oblique loph; 2: fused into a strongly oblique and thickened loph

1. m2-3 lingual branch of anterolophid

0: distinctly present; 1: very weak or absent

1. m2 sinusid

0: wide and transverse; 1: posteriorly directed; 2: anteriorly directed

1. m2 lingual dentine tract

0: smooth and far below the bottom of valleys; 1: slightly undulant, but far below the bottom of valleys; 2: undulant and nearly reaches or slightly overtops the bottom of valleys; 3: strongly undulant and intensively overtops the bottom of valleys

1. m3 lateral view

0: not oblique or curved; 1: slightly oblique; 2: strongly posterobuccally curved

1. m3 protosinusid

0: distinct; 1: very weak or absent

1. m3 posterosinusid

0: distinct and deep; 1: reduced and shallow

1. m3 lingual dentine tract

0: smooth and far below the bottom of valleys; 1: slightly undulant, but far below the bottom of valleys; 2: undulant and nearly reaches or slightly overtops the bottom of valleys

**Repository information about specimens described in this paper**

The following specimens described in this paper are housed in the Institute of Vertebrate Paleontology and Paleoanthropology (IVPP), Chinese Academy of Sciences, Beijing: Locality ZD1001, Zanda Basin, southwestern Xizang (Tibet) Autonomous Region: IVPP V 18032.1-29, 29 isolated teeth including 4 M1s, 5 M2s (2 broken), 3 M3s (1 broken), 6 m1s (1 broken), 6 m2s (1 broken) and 5 m3s (1 broken). All specimens collected are available for examination by qualified researchers.
